# Individual and household characteristics of persons with *Plasmodium falciparum* malaria in sites with varying endemicities in Kinshasa Province, Democratic Republic of the Congo

**DOI:** 10.1186/s12936-017-2110-7

**Published:** 2017-11-09

**Authors:** Melchior Kashamuka Mwandagalirwa, Lauren Levitz, Kyaw L. Thwai, Jonathan B. Parr, Varun Goel, Mark Janko, Antoinette Tshefu, Michael Emch, Steven R. Meshnick, Margaret Carrel

**Affiliations:** 1 Department of Epidemiology, University of North Carolina-Chapel Hill, CB7435, McGavran-Greenberg Hall, Chapel Hill, NC 27599 USA; 2 Ecole de Sante Publique, Faculte de Medecine, University of Kinshasa, Kinshasa, Democratic Republic of the Congo; 3 Division of Infectious Diseases, School of Medicine, University of North Carolina-Chapel Hill, 130 Mason Farm Road, Chapel Hill, NC 27599 USA; 4 Department of Geography, CB3220, University of North Carolina-Chapel Hill, Chapel Hill, NC 27599 USA; 5 Global Health Institute, Duke University, 229 Trent Hall, Durham, NC 27710 USA; 6 Department of Geographical & Sustainability Sciences, University of Iowa, 305 Jessup Hall, Iowa City, IA 52245 USA

**Keywords:** Malaria, Democratic Republic of the Congo, Longitudinal study, Surveillance, RDT, PCR

## Abstract

**Background:**

The Democratic Republic of the Congo (DRC) bears a large share of global malaria burden despite efforts to control and eliminate the disease. More detailed understanding of individual and household level characteristics associated with malaria are needed, as is an understanding of how these characteristics vary spatiotemporally and across different community-level malaria endemicities. An ongoing study in Kinshasa Province is designed to address gaps in prior malaria surveillance in the DRC by monitoring malaria across seasons, age groups and in high and low malaria sites. Across seven sites, 242 households and 1591 individuals are participating in the study. Results of the enrollment questionnaire, rapid diagnostic tests and PCR testing of dried blood spots are presented.

**Results:**

Overall malaria prevalence in the study cohort is high, 27% by rapid diagnostic test and 31% by polymerase chain reaction, and malaria prevalence is highly varied across very small geographic distances. Malaria prevalence is highest in children aged 6–15. While the majority of households own bed nets, bed net usage is less than 50%.

**Conclusions:**

The study cohort will provide an understanding of how malaria persists in populations that have varying environmental exposures, varying community-level malaria, and varying access to malaria control efforts.

**Electronic supplementary material:**

The online version of this article (10.1186/s12936-017-2110-7) contains supplementary material, which is available to authorized users.

## Background

Despite recent advances in malaria control efforts, malaria remains a major health problem in sub-Saharan Africa countries; 90% of the world’s malaria cases in 2015 were in the African region of the World Health Organization (WHO) [1]. In 2015, 9% of the world’s malaria deaths occurred in the Democratic Republic of the Congo (DRC) [1]. According to the 2016 World Malaria Report, 97% of the DRC’s 77 million people live in high risk zones [1]. However, although the malaria burden in the DRC is one of the highest in the world, spatial analysis of molecular malaria epidemiological studies have indicated that malaria in the DRC is highly spatially variable [2].

The prevalence of malaria in the DRC is over 30% in both adults and children; however, it is relatively understudied [2–10]. Furthermore, recent studies suggest that older children (ages 5–15) have high malaria burdens and may be an important malaria reservoir, but they are often excluded from surveys [11–15]. A cross-sectional study in the DRC recently showed that asymptomatic subjects of all ages carried *Plasmodium falciparum* [9]. However, further work is needed to assess the contribution of different age groups to the reservoir.

Malaria control and elimination efforts in the DRC, and elsewhere, focus on the distribution and usage of insecticide treated bed nets, indoor residual spraying of insecticides, artemisinin-based combination therapy (ACT), and rapid diagnostic tests (RDTs) in order to increase timely and effective treatment of infections. However, in the context of malaria elimination, while malaria prevalence continues to decline, it is necessary to target asymptomatic individuals who may carry gametocytes [16–22]. While little is known about the gametocytaemia reservoir(s) in the DRC, even less is known about what group would play an important role of transmission, given potentially varying levels of gametocytaemia. Consistent and longitudinal data on gametocyte carriage levels across age groups are needed to develop epidemiological models of transmission for malaria control and elimination purposes.

To address issues of limited malaria data collection in the DRC outlined above, the first prospective longitudinal study in the DRC to measure malaria prevalence and the gametocytaemia reservoir over time across varying levels of endemicity with subjects of all ages is being conducted. The study is also designed to examine potential for seasonal malaria fluctuation, with study households visited in both dry and rainy season, and to account for the potential of RDTs to fail to capture malaria infections. The baseline results of the study are presented here, with descriptive maps and statistics of the study population and malaria prevalence in individuals and households for both RDT and PCR detected malaria. Details on study design and administration and variations in malaria prevalence in rural and urban sites, across age categories and in relation to individual behaviours and household environmental exposures at enrollment are provided.

## Methods

### Study design

The DRC Health Monitoring Information System maintains a database on both malaria case counts and total population at the level of the health area. Health areas (Aire de Santé) are the smallest administrative units used for health surveillance by the DRC government. In Kinshasa Province, the 406 health areas are nested inside 35 health zones. The national health surveillance database was used to calculate the prevalence of malaria in each health area from two selected health zones in Kinshasa Province, Maluku and Lingwala, for the year 2014. These two health zones were selected based on accessibility year-round by the research team and to cover the urban/rural gradient. Health area prevalence estimates were partitioned into quartiles and randomly selected one health area from the quartiles to ensure that sampling sites came from different levels of malaria endemicity. Two health areas (Bu and Kimpoko) were chosen from the Maluku 1 health zone and one health area was selected from Lingwala, for a total of three health areas. The Maluku health zone is a rural zone while the Lingwala health zone is located in metropolitan area of Kinshasa City.

**Fig. 1:**
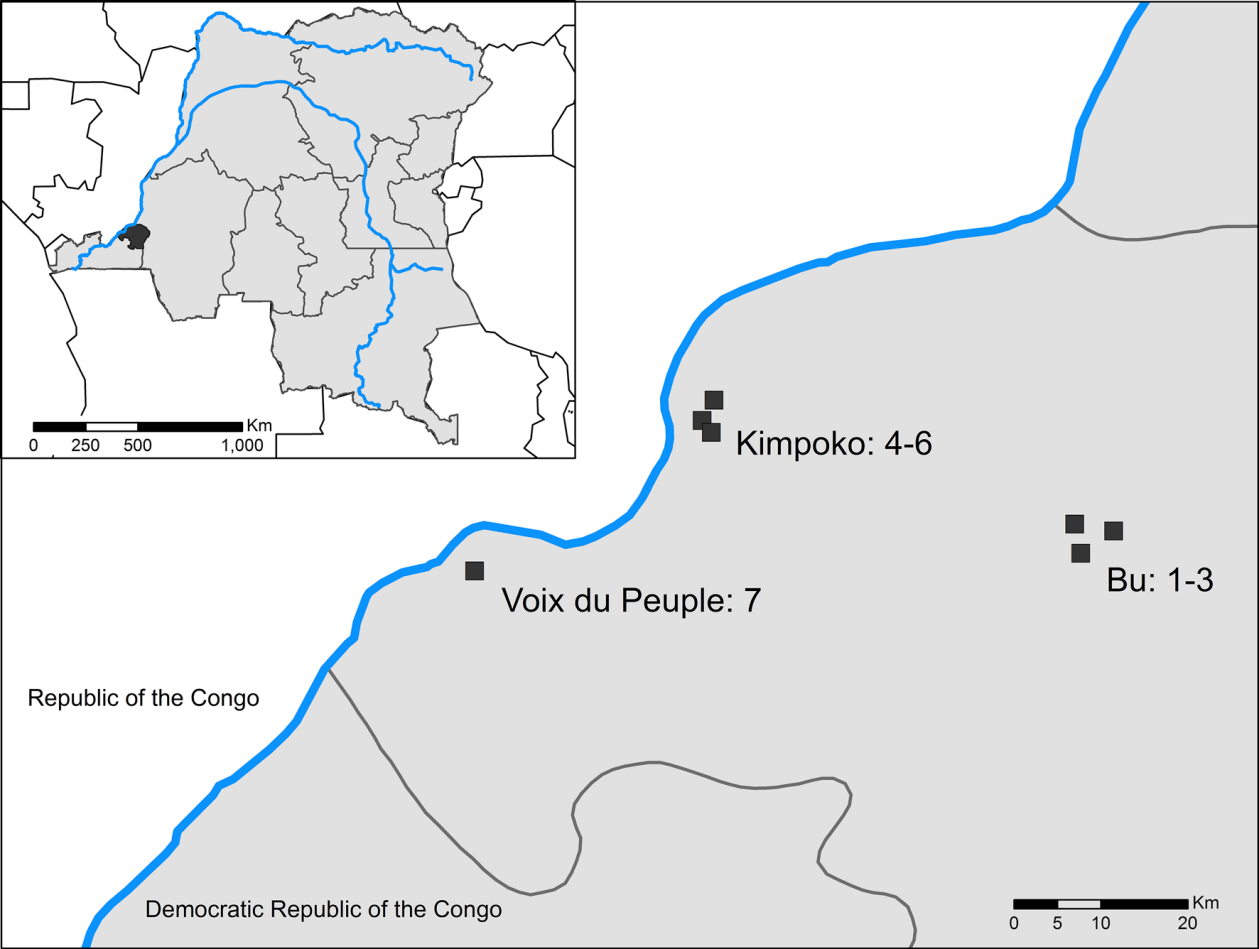
Location of Kinshasa Province within the Democratic Republic of the Congo (inset) and of study sites within the province

Health area maps were used in conjunction with Google Earth to identify all village sites within the selected two health areas of the Maluku 1 health zone. Three village sites within each health area were selected for inclusion in the study. Selected sites needed to meet the following eligibility criteria: (1) the households within each site were accessible by either vehicle or a short walk from the vehicle; (2) the population in each site was not transient; (3) there was an availability of sufficient numbers of individuals within the site to give informed consent. The Voix du Peuple health area located in the Lingwala health zone was chosen to provide a low malaria prevalence site of surveillance. Thus, seven sites with a range of malaria endemicities were selected for surveillance. These sites are located in Kinshasa Province of the DRC; three located in the interior of the province (#1–3 in the Bu health area), three near the Congo River (#4–6 in the Kimpoko health area), and one in the Kinshasa City metropolitan region (#7 in the Voix du Peuple health area) (Fig. 1).

For each of the seven surveillance locations, two geographic coordinates within the site were selected, a beginning and end point. Households were then randomly recruited, starting from the household nearest the start point coordinate and moving towards the household nearest the end point coordinate. Geographic coordinates of households that consented to participate in the study were recorded using GPS devices (Garmin Dakota 20 GPS). Study enrollment took place from February through May, 2015 during the rainy season.

The study team visited each randomly selected household to assess whether the household met enrollment criteria and to obtain informed consent, or parental assent for minors < 18 years of age, in Lingala and French. Informed consent and assent was given not only for collection of survey information but for collection and storage of biological specimens for future studies. The study population included both sexes, pregnant women and children, and complied with all pertinent ethical guidelines for studies in these populations. Individuals were eligible for inclusion if they had permanent residence within the study site and were willing and able to provide informed consent. Criteria for exclusion included evidence of impaired judgement for informed consent, an inability to understand one of the languages used by the study team, or serious concurrent illness. If a given household did not meet the inclusion criteria, it was replaced by the next closest household to the corresponding coordinates.

During the consent and assent process, each subject was told about the study, explaining that this would be a 2-year research study composed of both active and passive surveillance. For the 2-year study, each household would be visited by a study team two times per year for the active surveillance part of the study. One visit will occur in the dry season and one in the rainy season.

At the time of enrollment, the study team administered a questionnaire to each subject, requiring approximately 30 min. The questionnaire based upon the malaria module of the Demographic and Healthy Surveys (MEASURE-DHS, Rockville, MD), was administered. This questionnaire collects basic demographic information (sex, age) as well as information on bed net presence, age and usage (Additional file 1). Additional items in the questionnaire examine housing characteristics (construction materials), wealth (item ownership, electrification) and proximity to malaria risk factors (i.e. water). Residents also reported information on basic health outcomes related to malaria, such as having prior malaria diagnoses or the presence of fever.

In addition to completing the questionnaire, a fingerprick was performed on everyone ≥ 12 months of age and a heelprick on all children less than 1 year old at the baseline. The first 50 µl was applied to a SD Bioline Ag *P. falciparum* RDT (05FK60, Alere, Gyeonggi-do, Republic of Korea) and interpreted according to manufacturer’s protocol. The RDT was read on the spot. If positive, the subject was referred to the local health centre for treatment. After the baseline enrollment, the study team visited the local health centre to ascertain whether all RDT positive participants reached the clinic and accessed proper care and treatment. Additionally, community health workers responsible for the ongoing study surveillance were trained not only to recognize severe malaria symptoms as well as indications for ACT, but also to encourage participants to visit the health centre when sick and to follow the participant’s recovery at home after treatment. The study team, located in Kinshasa City, maintained contact with community health workers and the study clinic team to ensure that participants under ACT have recovered from malaria after the ACT ended. Any time a participant presented with severe malaria symptoms, he/she were referred to the study hospital for appropriate treatment. Participants received free ACT malaria treatment according to DRC government protocol; participants requiring treatment for severe malaria were provided free care by the study.

Subsequent to the collection of blood for the RDT, four dried blood spots (DBS), each containing approximately 50 µl of whole blood collected by finger-prick, was prepared on Whatman 3 MM filter paper (Fisher Scientific, Fair Lawn. NJ). The completed questionnaire and the filter paper containing the DBS were labelled with the subject’s code. After air-drying for at least 15 min or until dry at ambient temperature, the filter paper was placed in a ziplock bag-containing desiccant. DBS were then transported to the study office in Kinshasa and stored at – 20 °C until shipment to UNC-Chapel Hill for further testing.

In addition, at the time of enrollment, participants were told that any time they will experience fever or any symptoms related to malaria, they should visit the identified local study health centre for malaria diagnosis and treatment if needed. This is part of the passive surveillance of the study intended to estimate malaria incidence in the cohort. At the clinic during passive surveillance, all subjects had axillary temperature readings performed in addition to an RDT and hemoglobin. Even though microscopy was not part of the study design, any time smears were made and read, the results were recorded. Treatments given according to the National Malaria Programme guidelines were recorded.

Participants were instructed that whenever he/she subjectively experienced any adverse health event, or any other participant of his/her family experienced an adverse event, they were to report to the local health centre (clinic) or directly to the staff of the study. All adverse events, including malaria, will be reported to the study team in Kinshasa, and all serious events will be reported immediately to IRBs (KSPH and UNC).

At the end of the enrollment process, a study card was provided to participants in order to be presented anytime they visited a study clinic and during the subsequent household visits. This allows for the longitudinal, active and passive surveillance data to be accurately collected by clinic staff and community health workers. The completed questionnaire was labelled with the subject’s bar code and was subsequently double-entered and checked for consistency.

Subsequent to the enrollment data collection process, DNA was extracted from individual 6 mm DBS punches using Chelex as previously described [23]. The DNA was tested using a duplex real-time PCR assay targeting the *P. falciparum*-specific lactate dehydrogenase (*pfldh*) and human beta-tubulin (HumTuBB) genes, with a lower limit of detection of 5–10 parasites/µl (Additional file 2). All assays were performed in duplicate. Standards were made from cultured FCR3 strain *P. falciparum*, serially diluted in human whole blood after parasite density determination by three independent observers. The standards were prepared as 50 µl DBS using Whatman 3 MM filter paper and underwent DNA extraction using the same methods described above. Isolates that failed to amplify HumTuBB in duplicate or had a single positive replicate with cycle threshold value (C_T_) > 38 were excluded from analysis. Isolates were considered to be *pfldh* PCR positive if both replicates amplified or if a single replicate amplified with C_T_ ≤ 38, as previously described [24]. Before proceeding to data analysis, it was first confirmed that both replicates of standards containing 10 parasite/µl and greater successfully amplified. Regular quality control checks were performed to ensure consistent PCR performance across plates, with monitoring of mean C_T_ values, standard curve R^2^ values, and standard and negative control amplification by an independent observer, who also confirmed accurate entry of PCR results. While more sensitive PCR assays are available, a single-copy gene assay was chosen to improve the accuracy of the quantitative PCR results. Most single copy gene assays have limits of detection similar to the *pfldh* assay employed in this study. More sensitive assays typically target multi-copy genes or repetitive elements of the *P. falciparum* genome. While the presence of multiple targets allows for improved sensitivity, the number of gene copies can vary between parasites, making accurate quantitation impossible.

### Statistical methods

Only the baseline data collected at the time of enrollment is presented. Descriptive statistics at the individual and household level were generated. The relationship between demographic and behavioral characteristics and the presence of malaria parasites was assessed via Pearson’s Chi square test. The threshold for statistical significance was p = 0.05. A kappa statistic was calculated to determine concordance between RDT and PCR results for study individuals [25].

Rapid diagnostic test and PCR positive counts were generated at the household level as was the total population living in each household. To protect participant confidentiality and produce maps of malaria in the study sites, the total counts of RDT and PCR malaria and household populations were smoothed using kernel density estimation. In kernel density estimation a window (or kernel) estimates density at the window centroid using the values of all events (RDT or PCR or population counts) within the window [26]. Observations located closer to the window centroid are given greater weighting in the calculation of the density estimate. The kernel moves across the study area and calculates a density estimate for all locations in the seven study sites. The smoothed kernel for RDT and PCR was then divided by the kernel for population to generate an estimated surface of malaria prevalence in each of the study sites. A kernel of 100 m was used to capture heterogeneity in malaria prevalence across the small and compact study sites.

Chi square tests and calculation of the kappa statistic were completed in R (CRAN, R Foundation for Statistical Computing, Vienna, Austria). Mapping was completed in ArcGIS 10.4 (ESRI, Redlands, California).

## Results

1591 individuals in 242 households across the seven sites enrolled in the study. Among these 1591 individuals, 1559 DBS were used for PCR analysis. Of these, two were missing information from the malaria module of the questionnaire, resulting in an analytic dataset of 1557 individuals for PCR results and 1591 for the RDT results.

Of the 1591 individuals with RDT results, 427 (27%) tested positive for *P. falciparum* malaria. Of the 1557 participants included in the PCR analysis, 481 (31%) tested positive for *P. falciparum* malaria. Concordance between the RDT and PCR was substantial *(kappa* = 0.71, 95% CI 0.66–0.74*)*.

**Fig. 2:**
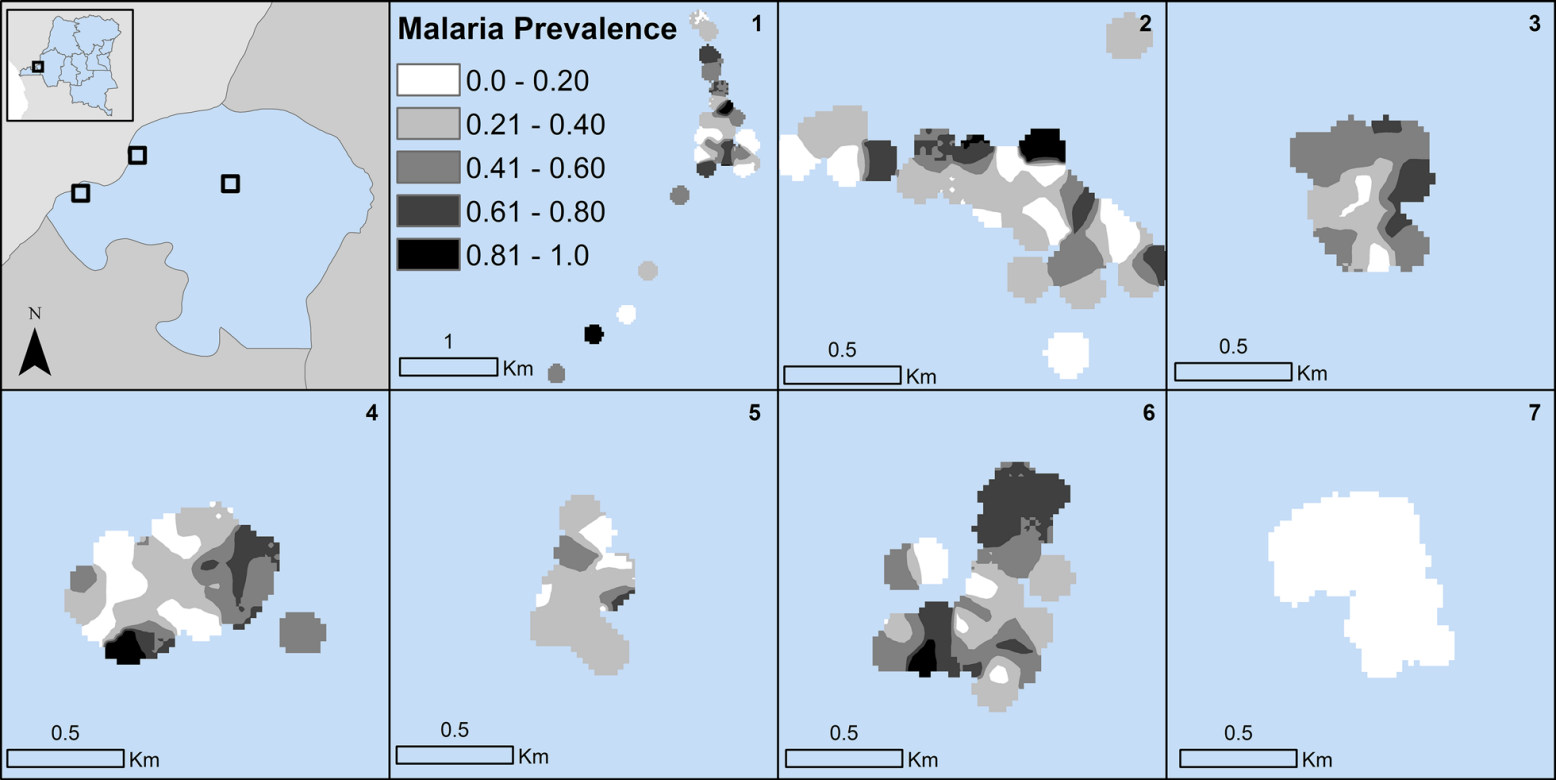
Kernel density estimates of PCR-positive malaria prevalence in the study sites

**Fig. 3:**
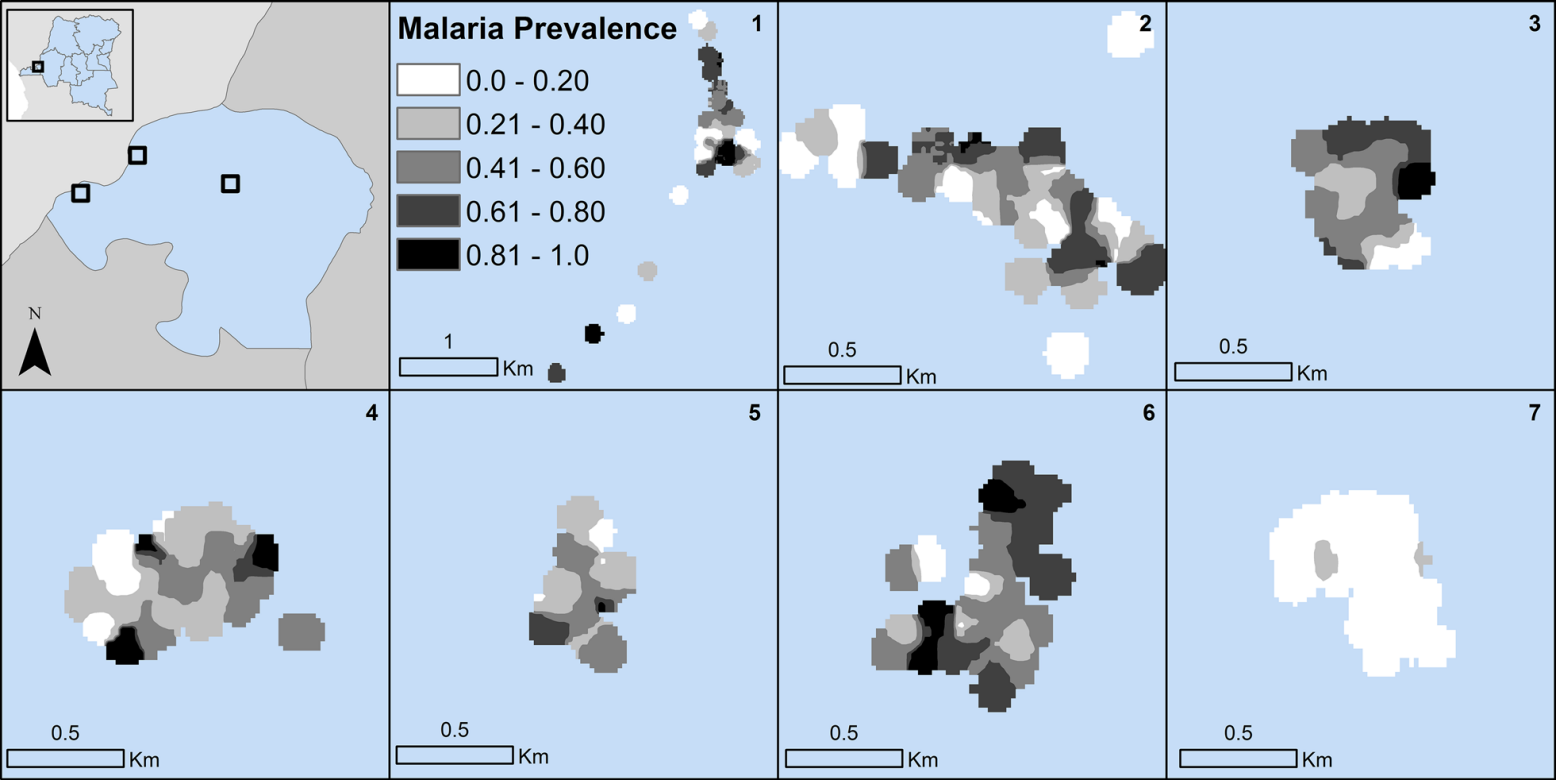
Kernel density estimates of PCR-positive malaria prevalence in the study sites

At the site scale, RDT-positive malaria prevalence ranged from a low of 3% in site 7 to a high of 42% in site 6 (median = 31%, p < 0.001) (Table 1). PCR-positive malaria prevalence exhibited a similar range, with the highest malaria prevalence in site 6 and lowest in site 7 and statistically significant Chi square differences between site-level prevalence. Site 7 is the only site located within urban Kinshasa. Within sites, malaria is not evenly distributed across households; ninety percent of all RDT and PCR malaria was observed in only 54% of the enrolled households, and approximately one quarter of households (RDT: n = 66, 27%; PCR: n = 62, 25.6%) had no malaria positive individuals at baseline. This heterogeneity in malaria prevalence across households is also observed in Figs. 2 and 3.

**Table 1:** Descriptive statistics for individual-level characteristics of participants

Variable	All	RDT+	RDT−	Χ^2^ p-value	Total	PCR+	PCR−	Χ^2^ p-value
Total	1591	427 (27%)	1164 (73%)		1557	481 (31%)	1076 (69%)	
Age								
< 1 year	59	9 (15%)	50 (85%)	*<* *0.001*	57	9 (16%)	48 (84%)	*<* *0.001*
1–5 years	307	89 (29%)	218 (71%)		299	79 (26%)	220 (74%)	
6–10 years	263	116 (44%)	147 (56%)		255	125 (49%)	130 (51%)	
11–15 years	232	97 (42%)	135 (58%)		229	108 (47%)	121 (53%)	
16–25 years	247	58 (24%)	189 (76%)		243	75 (31%)	168 (69%)	
> 25 years	483	58 (12%)	425 (88%)		474	85 (18%)	389 (82%)	
Sex								
Male	713	202 (28%)	511 (72%)	0.23	698	237 (34%)	461 (66%)	*0.02*
Female	878	225 (26%)	653 (74%)		859	244 (28%)	615 (72%)	
Malaria in previous 6 months								
Yes-once	278	77 (28%)	201 (72%)	*0.04*	275	71 (26%)	204 (74%)	*0.02*
Yes-many	114	43 (38%)	71 (62%)		112	46 (41%)	66 (59%)	
No	1195	306 (26%)	889 (74%)		1166	362 (31%)	804 (69%)	
NA	4	1 (25%)	3 (75%)		4	2 (50%)	2 (50%)	
Fever in previous week								
Yes	385	147 (38%)	238 (62%)	*<* *0.001*	380	151 (40%)	229 (60%)	*<* *0.001*
No	1201	278 (23%)	923 (77%)		1172	327 (28%)	845 (72%)	
NA	5	2 (40%)	3 (60%)		5	3 (60%)	2 (40%)	
Slept under bed net previous night								
Yes	714	194 (27%)	520 (73%)	*<* *0.001*	701	208 (30%)	493 (70%)	0.37
No	877	233 (27%)	644 (73%)		856	273 (32%)	583 (68%)	
Age of the bed net								
< 6 months	77	22 (29%)	55 (71%)	0.58	77	22 (29%)	55 (71%)	0.20
6 months–1 year	70	13 (19%)	57 (81%)		70	13 (19%)	57 (81%)	
1–3 years	339	97 (29%)	242 (71%)		329	103 (31%)	226 (68.7%)	
> 3 years	110	31 (28%)	79 (72%)		110	32 (29%)	78 (71%)	
No net or do not know	995	264 (26%)	731 (74%)		971	311 (32%)	660 (68%)	
Site of residence								
1	209	85 (41%)	124 (59%)	*<* *0.001*	205	95 (46%)	110 (54%)	*<* *0.001*
2	217	67 (31%)	150 (69%)		210	80 (38%)	130 (62%)	
3	249	91 (37%)	158 (63%)		241	101 (42%)	140 (58%)	
4	268	78 (29%)	190 (71%)		258	79 (31%)	179 (69%)	
5	92	25 (27%)	67 (73%)		92	32 (35%)	60 (65%)	
6	168	70 (42%)	98 (58%)		167	79 (47%)	88 (53%)	
7	388	11 (3%)	377 (97%)		384	15 (4%)	369 (96%)	

Statistically significant p-values are indicated in italic

Malaria prevalence varied considerably by age, with PCR prevalence ranging from 15.8% in children less than 1 and 17.9% in adults older than 25–49% in 6–10 year olds (p < 0.001). RDT-based diagnostics revealed similar results, with 15.3% in children less than 1 and 12% in adults older than 25–44% in 6–10 year olds (p < 0.001). Females in the study had lower prevalence of malaria than men.

**Fig. 4:**
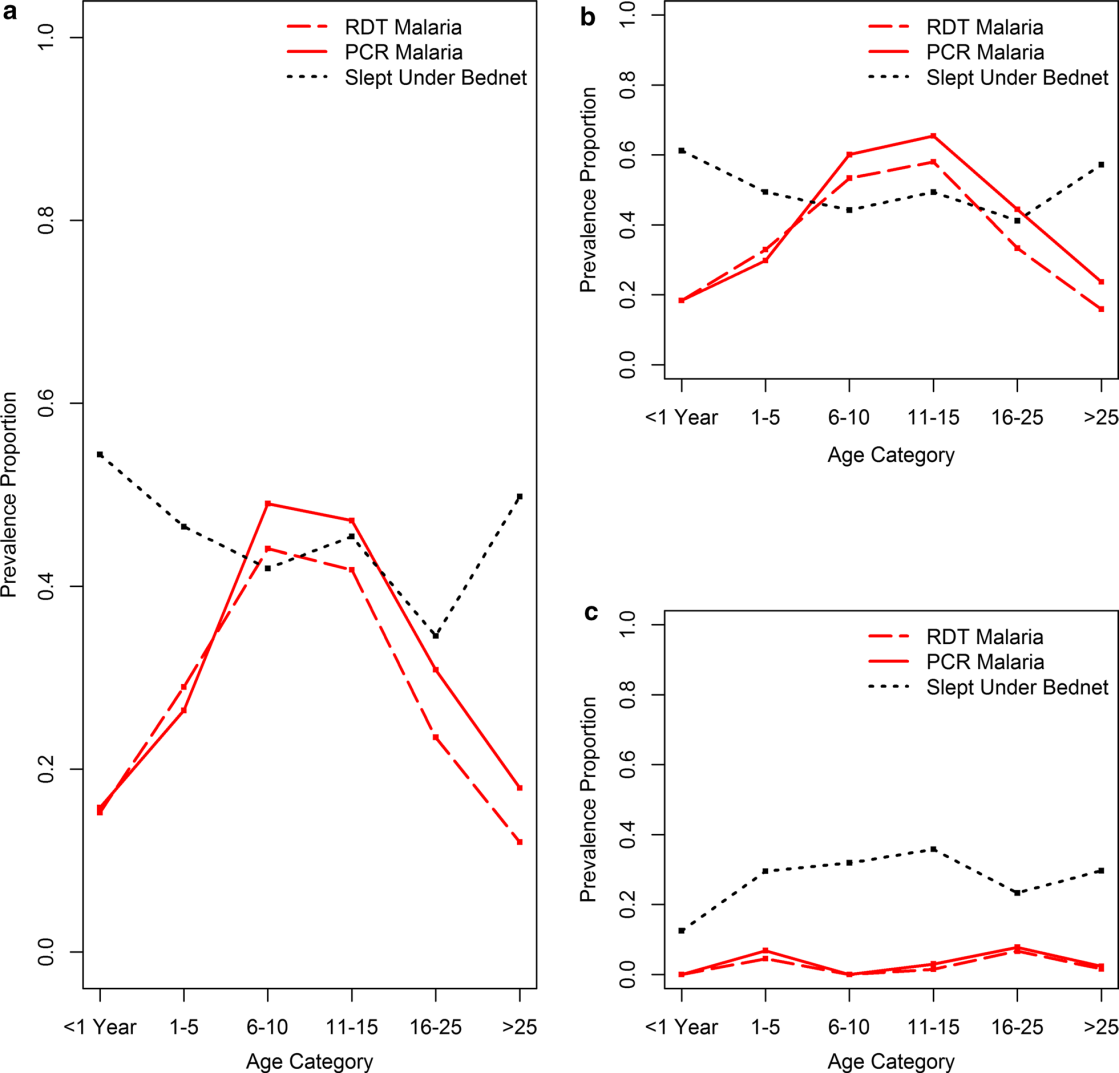
Prevalence of malaria by RDT and PCR and of bed net usage the night prior stratified by age category for all sites (**a**). Prevalence of malaria via RDT and PCR and of bed net usage stratified by age category for rural sites 1–6 (**b**) and urban site 7 (**c**). Note that prevalence is calculated among the members of each specific age class rather than the entire dataset

Fewer individuals with RDT-positive malaria at time of study enrollment reported fever in the previous week than those testing negative (38% vs 62%, p < 0.001). Similarly, only 40% of individuals with PCR-positive malaria reported fever in the previous week (p < 0.001). Individuals who reported prior malaria infections in the 6 months prior to study enrollment had higher malaria prevalence than those with no prior malaria infections, particularly among those who reported many prior malaria infections (12.1% higher RDT, p = 0.02; 10% higher PCR, p = 0.02) at the baseline enrollment. Individuals who reported sleeping under a bed net the previous night had slightly higher RDT-positive malaria prevalence and lower PCR-positive prevalence than those not sleeping under bed nets, though Chi square tests indicated these differences were insignificant. Bed net usage varies by age, with study participants in the lowest and oldest age categories reporting higher bed net usage the night prior (Fig. 4a). When site 7, the urban site with low malaria prevalence, is considered separately from the other six sites, differences in malaria prevalence and bed net usage by participant age are observed (Fig. 4b, c). All age groups in site 7 have malaria prevalence below eight percent (by both RDT and PCR), while in rural sites, malaria prevalence in sites 1–6 ranges from a low of 16% to a high of 65%. Bednet usage in site 7 peaks in children, the opposite pattern of bed net usage by age category observed in the other sites. Twenty-nine percent of individuals in site 7 (112/384) reported bed net usage the night prior to study enrolment, in contrast to 50% (589/1173) who slept under a bed net in sites 1–6. No significant differences in either RDT-positive or PCR-positive malaria outcomes are observed when bed nets are stratified by age of net.

**Fig. 5:**
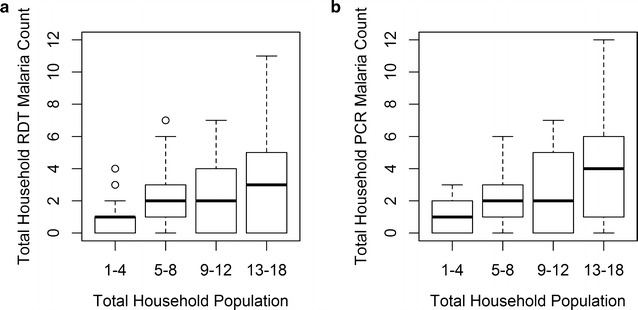
Malaria counts by RDT (**a**) and PCR (**b**) in households stratified by household population size

Household size varied greatly within the study population, from single individual households to 18 member households (median = 7 persons) (Table 2). Households with larger numbers of enrolled participants were associated with a wider range of observed malaria (Fig. 5). Individuals living in households with glass and screen windows had lower prevalence of RDT and PCR malaria than those without. Households with open windows had higher and significantly different malaria prevalence than those without open windows for both RDT and PCR outcomes. Malaria prevalence for individuals living in households with electricity was lower and significantly different in both the RDT and PCR datasets. Malaria prevalence was similar among individuals who resided in households that owned at least one bed net versus residence in a household with no bed net for both RDT and PCR outcomes. Seventy-five percent of individuals in sites 1–6 (874/1173) reside in a household that owns a bed net. In contrast, only sixty-six percent of individuals (254/384) in site 7 live in a household with a bed net.

**Table 2:** Descriptive statistics for household-level characteristics of participants

	Total	RDT+	RDT−	Χ^2^ p-value	Total	PCR+	PCR−	Χ^2^ p-value
Total household population								
1–4	188	61 (33%)	127 (67%)	*0.013*	194	66 (34%)	128 (66%)	*0.014*
5–8	782	226 (29%)	556 (71%)		771	261 (34%)	510 (66%)	
9–12	410	91 (22%)	319 (78%)		368	96 (26%)	272 (74%)	
13–18	211	49 (23%)	162 (77%)		224	58 (26%)	166 (74%)	
Glass windows								
Yes	207	5 (2%)	202 (98%)	*<* *0.001*	203	9 (4%)	194 (96%)	*<* *0.001*
No	1384	422 (31%)	962 (69%)		1354	472 (35%)	882 (65%)	
Screen windows								
Yes	44	5 (11%)	39 (89%)	*0.029*	44	5 (11%)	39 (89%)	*0.007*
No	1547	422 (27%)	1125 (73%)		1513	476 (32%)	1037 (68%)	
Open windows								
Yes	285	105 (37%)	180 (63%)	*<* *0.001*	278	122 (44%)	156 (56%)	*<* *0.001*
No	1306	322 (25%)	984 (75%)		1279	359 (28%)	920 (72%)	
Electricity								
Yes	388	11 (3%)	377 (97%)	*<* *0.001*	384	15 (4%)	369 (96%)	*<* *0.001*
No	1203	416 (35%)	787 (65%)		1173	466 (40%)	707 (60%)	
Household owns bed net								
Yes	1152	313 (27%)	839 (73%)	0.671	1128	344 (31%)	784 (69%)	0.626
No	439	114 (26%)	325 (74%)		429	137 (32%)	292 (68%)	
Water in 2 min								
Yes	632	163 (26%)	469 (74%)	0.479	616	186 (30%)	430 (70%)	0.67
No	959	264 (27%)	695 (73%)		941	295 (31%)	646 (69%)	
Type of water								
Stream	98	33 (34%)	65 (66%)	0.081	98	39 (40%)	59 (60%)	*0.002*
Pond/lake	39	12 (31%)	27 (69%)		38	13 (34%)	25 (66%)	
Swamp/marsh	57	22 (39%)	35 (61%)		57	28 (49%)	29 (51%)	
Frequent puddles	264	74 (28%)	190 (72%)		254	85 (34%)	169 (66%)	
NA/other	1133	286 (25%)	847 (75%)		1110	316 (29%)	794 (71%)	

Statistically significant p-values are indicated in italic

Malaria prevalence was also similar among those living within 2 min of a water source compared to individuals living more than 2 min away for both RDT and PCR. When water type was stratified, however, RDT prevalence ranged from 25 to 39% and PCR prevalence ranged from 29 to 49%, with the highest prevalence of malaria for both outcomes observed in individuals with residential proximity to a swamp or marsh.

## Discussion

Overall malaria prevalence in the population enrolled in the study is high, 26.8% for RDT and 30.9% for PCR, though this varies significantly by site. Site 7, located in urban Kinshasa, has much lower malaria prevalence (2.8%) than the sites located in more rural areas of the province. Other research has suggested lower malaria within Kinshasa than its surrounding peri-urban region [27]. Within sites, there is high variance in malaria prevalence across short distances, a phenomenon that has been observed elsewhere (Figs. 2 and 3) [28–33]. Similarly, a little over half (54%) of households are responsible for a majority (90%) of the malaria cases in the study population.

The high spatial variation over short distances might be explained by variation in not only individual and household characteristics but also heterogeneity in environmental factors that can result in greater malaria outcomes [29, 32, 34]. While malaria prevalence did not vary significantly when stratified by household proximity to any type of water, approximately 25% for RDT-positive and 30% for PCR-positive individuals in households both near and far from water sources, there was a significant range in malaria prevalence when the type of water source was the categorical definition. Positive malaria tests are highest in individuals living in close proximity to marshes and swamps (38.6% RDT and 49.1% PCR). Others have found that landscape features conducive to mosquito breeding and biting, and associated with risk of malaria to individuals, shift seasonally [35, 36]. While baseline results are presented in this paper, the longitudinal data collection that is ongoing in this study population will allow future examination of how malaria varies not only spatially but also temporally.

Concordance between PCR and RDT results is high but not absolute; approximately 12% of samples were discordant, and the kappa statistic of 0.706 indicates good agreement. This discordance results in slight variation in the relationship between individual and household level characteristics and malaria outcomes. In the RDT dataset, for instance, malaria in females is only 2.7% lower than in males (p = 0.23) while in the PCR dataset the 5.5% lower malaria observed in females is significantly different than malaria in males (p = 0.02). Some degree of discordance is expected due to differences in the sensitivity of the diagnostic tests; the PCR assays employed have limits of detection > 10-fold lower than RDTs. Additionally, because PCR is better suited for detection of low parasitaemia infections, asymptomatic infections can be more frequently detected by PCR than RDT. Future research will explore other reasons for discordant results, including *pfhrp2/3*-deleted *P. falciparum* and/or spatial heterogeneity of the subpatent reservoir [37].

The majority of households in the study own a bed net (174/242, 72%), though less than half of participants reported sleeping under a bed net the night prior (~ 45% in both RDT and PCR datasets). No significant differences in malaria counts were observed when individuals were stratified by their usage of bed nets. While in the overall dataset there was variation of only 1–2% in malaria outcomes when individuals were stratified by bed net use the night prior to enrollment, we observe variation in this relationship when the age of participants is taken into consideration (Fig. 4). Bed net usage is highest in children under the age of 1 (54%) and falls in older children and young adults to a low of 34% for individuals in their early 20 s. Bed net usage then increases in ages greater than 25. Other studies have shown that malaria prevalence is higher in children and young adults, and also that characteristics of the bed nets themselves, such as the age of the bed net, impact not only bed net efficacy but also likelihood of use [11–13, 15, 38]. The age profile of malaria positive individuals in the baseline data for the high malaria prevalence sites 1–6 indicate the same, with highest prevalence in children and teenagers, and that these age categories report lower bed net usage. The findings from this study, however, do not suggest variation in malaria prevalence when characteristics of the bed nets, such as the age of the bed net, is taken into consideration.

Large differences in malaria outcomes are observed when households are categorized by their physical features, with lower malaria prevalence in individuals residing in households with glass or screen windows and electricity. However, electricity was reported only in site 7, located in Kinshasa City, and the majority of households with glass and screen windows were also in this location. When data from site 7 are excluded, there are no significant differences in malaria outcomes for individuals living in households with screens or glass windows. Thus, while glass, screens and electricity provide not only physical barriers to mosquito entry into the household they also serve as a marker of wealth and, in this case, urbanicity, and suggest that it is the urban environment of Kinshasa City that is protective rather than the physical features of the household. Though not all of this site’s (site 7) enrolled participants had these household characteristics, their overall higher prevalence may result in protection to both the individual and the community (“the herd”) and be responsible for the much lower prevalence of malaria (2.8% via RDT and 3.9% via PCR) in this site in comparison to others. This herd protection also potentially explains the low malaria prevalence in site 7 despite lower bed net ownership and lower bed net usage than in the rural, high malaria sites.

## Conclusion

The findings described represent the baseline results of ongoing active and passive surveillance of malaria outcomes in a diversity of settings within Kinshasa Province, Democratic Republic of the Congo. The spatial, epidemiological, and diagnostic data obtained during this longitudinal study are intended to support malaria policy decisions in the DRC and serve as a valuable resource for future research.

## Additional files

**Additional file 1.** Baseline survey.

**Additional file 2: Table S1.** PCR Primers.
