# Correction to: Individual and household characteristics of persons with *Plasmodium falciparum* malaria in sites with varying endemicities in Kinshasa Province, Democratic Republic of the Congo

**DOI:** 10.1186/s12936-018-2429-8

**Published:** 2018-08-08

**Authors:** Melchior Kashamuka Mwandagalirwa, Lauren Levitz, Kyaw L. Thwai, Jonathan B. Parr, Varun Goel, Mark Janko, Antoinette Tshefu, Michael Emch, Steven R. Meshnick, Margaret Carrel

**Affiliations:** 1 Department of Epidemiology, University of North Carolina-Chapel Hill, CB7435, McGavran-Greenberg Hall, Chapel Hill, NC 27599 USA; 2 Ecole de Sante Publique, Faculte de Medecine, University of Kinshasa, Kinshasa, Democratic Republic of the Congo; 3 Division of Infectious Diseases, School of Medicine, University of North Carolina-Chapel Hill, 130 Mason Farm Road, Chapel Hill, NC 27599 USA; 4 Department of Geography, CB3220, University of North Carolina-Chapel Hill, Chapel Hill, NC 27599 USA; 5 Global Health Institute, Duke University, 229 Trent Hall, Durham, NC 27710 USA; 6 Department of Geographical & Sustainability Sciences, University of Iowa, 305 Jessup Hall, Iowa City, IA 52245 USA

## Correction to: Malar J (2017) 16:456 10.1186/s12936-017-2110-7

Unfortunately after publication of the original article [1], it came to the author’s attention that there is an error in the caption of Fig. 2.

Figure 2 is incorrectly captioned as: “Kernel density estimates of PCR-positive malaria prevalence in the study sites”.

Please be advised that the correct caption for Fig. 2 is “Fig. 2. Kernel density estimates of RDT-positive malaria prevalence in the study sites”.

## References

[1] Mwandagalirwa MK, Levitz L, Thwai KL, Parr JB, Goel V, Janko M, Tshefu A, Emch M, Meshnick SR, Carrel M (2017). Individual and household characteristics of persons with *Plasmodium falciparum* malaria in sites with varying endemicities in Kinshasa Province, Democratic Republic of the Congo. Malar J.

