# Supplementary material for: Individual and household characteristics of persons with Plasmodium falciparum malaria in sites with varying endemicities in Kinshasa Province, Democratic Republic of the Congo
Source: Malar J. 2017 Nov 9;16:456. doi: 10.1186/s12936-017-2110-7 (PMC5680818; doi:10.1186/s12936-017-2110-7)
Supplement: Supplementary file 1 — Additional file 1. Baseline survey. [file 12936_2017_2110_MOESM1_ESM.pdf]

Baseline Household Questionnaire. *(To be completed by the interviewer.)*

Date of Visit: |__|__| - |__|__| - |__|__|__|__|
day month year

Endemicity ID (*circle one*): | 01 | 02 | 03 | 04 |

Village ID: [ ][ ]

Household ID: [ ][ ]

X Coord: [ ][ ][ ][ ][ ][ ][ ]

Y Coord: [ ][ ][ ][ ][ ][ ][ ]

Household characteristics:

1. What is the material used for construction of this house? *Circle all that apply*.

Roof material:

No roof	00	Palm leaves/bamboo	04	Wood	08	Shingles	12
Thatching/leaves	01	Wooden planks	05	Zinc/cement fiber	09	Other	97
Earth clods	02	Cardboard	06	Tiles	10	Don't know	98
Mats	03	Sheet metal	07	Concrete (cement)	11	Refused	99

If other, please list: ____________________

Outside wall of house:

No walls	00	Stones with mud	04	Cement	09	Wooden planks/shingles	14
Earth	01	Uncovered adobe	05	Stones with cement/lime	10	Other	97
Bamboo/cane/ palms/tree trunks	02	Plywood	06	Bricks	11	Don't know	98
		Cardboard	07	Cement blocks	12	Refused	99
Bamboo with mud	03	Reclaimed wood	08	Covered adobe	13		

If other, please list: ____________________

Floor material:

Earth/sand	01	Bamboo/palm leaves	04	Tiles	07	Other	97
Dung	02	Parquet/polished wood	05	Cement	08	Don't know	98
Wooden planks	03	Vinyl strips/asphalt	06	Carpet	09	Refused	99

If other, please list: ____________________

Windows:

No windows	00	Open	03	Planks	05	Don't know	98
Glass	01	Plastic paper/carton	04	Other	97	Refused	99
Screens	02						

If other, please list: ____________________

2. Are there visible holes, cracks, or open eaves in the walls of the house?

No	00	Yes	01	Don't know	98	Refused	99

3. In this household, do you have (*circle all that apply*):

Electricity	01	A refrigerator	05	One or more beds	08	A sewing machine	12
A toilet	02	A gas or electric stove/ cooktop	06	One or more lamps	09	Don't know	98
A radio	03			An oven	10	Refused	99
A TV	04	A generator	07	One or more hoes	11		

4. Is there someone in this household who has (*circle all that apply*):

A watch	01	A motorcycle or scooter	04	A motorboat	07	A house for rent	10
A cell phone	02	A car or truck	05	A bicycle	08	Don't know	98
A whaleboat/motorized canoe	03	A cart pulled by an animal	06	A computer	09	Refused	99

5. Does someone in your household own cultivable land?

No	00	Yes	01	Don't know	98	Refused	99

5a. If yes, how many hectares of cultivable land do they own? [ ][ ] hectares
If 0, enter 00. If ≥95, enter 95. If N/A, enter 96. If don't know, enter 98. If refused, enter 99.

Interviewer Code: ________________

Baseline Household Questionnaire. *(Continued)*

Endemicity-Village-Household ID: ☐☐☐☐☐☐

01	02	03	04	05	06	07	08	09	10	11
Line #	List the names of people who usually live in this household and visitors who spent last night here, starting with the head of the house.	What is the relationship of (NAME) to the head of the house?<br>See codes below.	Is (NAME) male or female?	Does (NAME) usually live in this house?	Did (NAME) spend last night here?	What age is (NAME)?<br>If <5 years, enter age in months.<br>Circle years or months for age as appropriate.<br>If ≥95 years, enter 95.<br>If don't know, enter 98.<br>If refused, enter 99.	If age ≥15, what is the marital status of (NAME)?<br>00=Never married or lived together<br>01=Married/live together<br>02=Divorced/ separated<br>03=Widowed<br>96=N/A<br>99=Refused	If age ≥5, what is the highest level of education (NAME) has completed?<br>See codes below.	If age ≥15, what is the usual occupation of (NAME)?<br>See codes below.	What religion does (NAME) identify with?<br>See codes below.
01		☐☐	F M<br>1 0	Y N<br>1 0	Y N<br>1 0	☐☐ Years / Months	☐☐	☐☐	☐☐	☐☐
02		☐☐	1 0	1 0	1 0	☐☐ Years / Months	☐☐	☐☐	☐☐	☐☐
03		☐☐	1 0	1 0	1 0	☐☐ Years / Months	☐☐	☐☐	☐☐	☐☐
04		☐☐	1 0	1 0	1 0	☐☐ Years / Months	☐☐	☐☐	☐☐	☐☐
05		☐☐	1 0	1 0	1 0	☐☐ Years / Months	☐☐	☐☐	☐☐	☐☐
06		☐☐	1 0	1 0	1 0	☐☐ Years / Months	☐☐	☐☐	☐☐	☐☐
07		☐☐	1 0	1 0	1 0	☐☐ Years / Months	☐☐	☐☐	☐☐	☐☐
08		☐☐	1 0	1 0	1 0	☐☐ Years / Months	☐☐	☐☐	☐☐	☐☐
09		☐☐	1 0	1 0	1 0	☐☐ Years / Months	☐☐	☐☐	☐☐	☐☐
10		☐☐	1 0	1 0	1 0	☐☐ Years / Months	☐☐	☐☐	☐☐	☐☐

Code for Q3:

00 = Unrelated
01 = Head of house
02 = Spouse
03 = Son or daughter
04 = Step-son/daughter
05 = Grandson/daughter
06 = Father or mother
07 = In-laws
08 = Brother or sister
09 = Nephew or niece
10 = Nephew/niece by marriage
11 = Child adopted by/in custody of head of house
97 = Other
98 = Don't know
99 = Refused

Code for Q9:

00 = No school
01 = Some primary school
02 = Primary school certificate
03 = 1st orientation cycle
04 = 2nd orientation cycle
05 = 3rd year secondary school
06 = 4th year secondary school
07 = 5th year secondary school
08 = 6th year secondary school
09 = 3-year university
10 = 5-year university
11 = Doctorate
96 = N/A
97 = Other
98 = Don't know
99 = Refused

Code for Q10:

00 = No employment
01 = State employee
02 = Soldier or policeman
03 = Private company employee
04 = Farmer
05 = Fisherman
06 = Driver
07 = Hotel worker
08 = Road cleaner
09 = Market seller
10 = Boutique owner
11 = Street vendor
12 = Money changer
13 = Artist
14 = Tailor
15 = Small personal business
16 = Student
96 = N/A
97 = Other
98 = Don't know
99 = Refused

Code for Q11:

00 = None
01 = Traditional religion
02 = Catholic
03 = Evangelical
04 = The Awakening Church
05 = Adventist
06 = Protestant
07 = Muslim
97 = Other
98 = Don't know
99 = Refused

Interviewer Code: ________________

Baseline Household Questionnaire. *(Continued)*

Endemicity-Village-Household ID: [ ][ ][ ][ ][ ][ ]

01	02	03	04	05	06	07	08	09	10	11
Line #	List the names of people who usually live in this household and visitors who spent last night here, starting with the head of the house.	What is the relationship of (NAME) to the head of the house?<br>See codes below.	Is (NAME) male or female?	Does (NAME) usually live in this house?	Did (NAME) spend last night here?	What age is (NAME)?<br>If <5 years, enter age in months.<br>Circle years or months for age as appropriate.<br>If ≥95 years, enter 95.<br>If don't know, enter 98.<br>If refused, enter 99.	If age ≥15, what is the marital status of (NAME)?<br>00=Never married or lived together<br>01=Married/live together<br>02=Divorced/ separated<br>03=Widowed<br>96=N/A<br>99=Refused	If age ≥5, what is the highest level of education (NAME) has completed?<br>See codes below.	If age ≥15, what is the usual occupation of (NAME)?<br>See codes below.	What religion does (NAME) identify with?<br>See codes below.
			F M	Y N	Y N					
11		[ ][ ]	1 0	1 0	1 0	[ ][ ] Years Months	[ ][ ]	[ ][ ]	[ ][ ]	[ ][ ]
12		[ ][ ]	1 0	1 0	1 0	[ ][ ] Years Months	[ ][ ]	[ ][ ]	[ ][ ]	[ ][ ]
13		[ ][ ]	1 0	1 0	1 0	[ ][ ] Years Months	[ ][ ]	[ ][ ]	[ ][ ]	[ ][ ]
14		[ ][ ]	1 0	1 0	1 0	[ ][ ] Years Months	[ ][ ]	[ ][ ]	[ ][ ]	[ ][ ]
15		[ ][ ]	1 0	1 0	1 0	[ ][ ] Years Months	[ ][ ]	[ ][ ]	[ ][ ]	[ ][ ]
16		[ ][ ]	1 0	1 0	1 0	[ ][ ] Years Months	[ ][ ]	[ ][ ]	[ ][ ]	[ ][ ]
17		[ ][ ]	1 0	1 0	1 0	[ ][ ] Years Months	[ ][ ]	[ ][ ]	[ ][ ]	[ ][ ]
18		[ ][ ]	1 0	1 0	1 0	[ ][ ] Years Months	[ ][ ]	[ ][ ]	[ ][ ]	[ ][ ]
19		[ ][ ]	1 0	1 0	1 0	[ ][ ] Years Months	[ ][ ]	[ ][ ]	[ ][ ]	[ ][ ]
20		[ ][ ]	1 0	1 0	1 0	[ ][ ] Years Months	[ ][ ]	[ ][ ]	[ ][ ]	[ ][ ]

Code for Q3:

00 = Unrelated
01 = Head of house
02 = Spouse
03 = Son or daughter
04 = Step-son/daughter
05 = Grandson/daughter
06 = Father or mother
07 = In-laws
08 = Brother or sister
09 = Nephew or niece
10 = Nephew/niece by marriage
11 = Child adopted by/in custody of head of house
97 = Other
98 = Don't know
99 = Refused

Code for Q9:

00 = No school
01 = Some primary school
02 = Primary school certificate
03 = 1st orientation cycle
04 = 2nd orientation cycle
05 = 3rd year secondary school
06 = 4th year secondary school
07 = 5th year secondary school
08 = 6th year secondary school
09 = 3-year university
10 = 5-year university
11 = Doctorate
96 = N/A
97 = Other
98 = Don't know
99 = Refused

Code for Q10:

00 = No employment
01 = State employee
02 = Soldier or policeman
03 = Private company employee
04 = Farmer
05 = Fisherman
06 = Driver
07 = Hotel worker
08 = Road cleaner
09 = Market seller
10 = Boutique owner
11 = Street vendor
12 = Money changer
13 = Artist
14 = Tailor
15 = Small personal business
16 = Student
96 = N/A
97 = Other
98 = Don't know
99 = Refused

Code for Q11:

00 = None
01 = Traditional religion
02 = Catholic
03 = Evangelical
04 = The Awakening Church
05 = Adventist
06 = Protestant
07 = Muslim
97 = Other
98 = Don't know
99 = Refused

Interviewer Code: ________________

Endemicity-Village-Household ID: | | | | | | |

6. Is there water within a two-minute walk from your house?

No 00	Yes 01	Don't know 98	Refused 99

6a. If yes, what type of water is it?

Stream 01	Pond/lake 02	Swamp/ marsh 03	Frequent puddles 04	N/A 96	Other 97	Don't know 98	Refused 99

If other, please list: ____________________________

7. Does your household have mosquito nets for sleeping?

No 00	Yes 01	Don't know 98	Refused 99

6a. If yes, how many mosquito nets do you have in your house? | | | nets

If ≥7, enter 07.
If N/A, enter 96.
If don't know, enter 98.
If refused, enter 99.

IF HOUSEHOLD DOES NOT HAVE MOSQUITO NETS FOR SLEEPING: skip to page 7.

Interviewer Code: _________________

Baseline Household Questionnaire. *(Continued)*

Endemicity-Village-Household ID: ☐☐☐☐☐☐

#	Bed Net Questions	Net #1	Net #2	Net #3
01	INTERVIEWER RESPONDS: Did you observe this net?	Observed 01<br>Not observed 00	Observed 01<br>Not observed 00	Observed 01<br>Not observed 00
02	INTERVIEWER RESPONDS: Are there holes in this net?	Yes 01<br>No 00<br>N/A 96	Yes 01<br>No 00<br>N/A 96	Yes 01<br>No 00<br>N/A 96
03	INTERVIEWER RESPONDS: Is this net correctly installed?	Yes 01<br>No 00<br>N/A 96	Yes 01<br>No 00<br>N/A 96	Yes 01<br>No 00<br>N/A 96
04	How many months have you had this net?<br>If <1 month, enter 00.<br>If >36 months, enter 95.<br>If don't know, enter 98.<br>If refused, enter 99.	☐☐ months	☐☐ months	☐☐ months
05	Where did you get this net?	Purchased at market 01<br>Mass distribution 02<br>Antenatal clinic 03<br>Hospital 04<br>N/A 96<br>Other 97<br>*Please list:* ________<br>Don't know 98<br>Refused 99	Purchased at market 01<br>Mass distribution 02<br>Antenatal clinic 03<br>Hospital 04<br>N/A 96<br>Other 97<br>*Please list:* ________<br>Don't know 98<br>Refused 99	Purchased at market 01<br>Mass distribution 02<br>Antenatal clinic 03<br>Hospital 04<br>N/A 96<br>Other 97<br>*Please list:* ________<br>Don't know 98<br>Refused 99
06	Did someone sleep under this net last night?<br>**IF NO: return to Question 1 for the next net or skip to page 7 if this is the last net.**	Yes 01<br>No 00<br>N/A 96<br>Don't know 98<br>Refused 99	Yes 01<br>No 00<br>N/A 96<br>Don't know 98<br>Refused 99	Yes 01<br>No 00<br>N/A 96<br>Don't know 98<br>Refused 99
06a	If yes to Question 6: who slept under this net?<br>Enter the name and age of each person.	Name / Age<br>Person 1 ☐ ☐<br>Person 2 ☐ ☐<br>Person 3 ☐ ☐<br>Person 4 ☐ ☐<br>Person 5 ☐ ☐	Name / Age<br>Person 1 ☐ ☐<br>Person 2 ☐ ☐<br>Person 3 ☐ ☐<br>Person 4 ☐ ☐<br>Person 5 ☐ ☐	Name / Age<br>Person 1 ☐ ☐<br>Person 2 ☐ ☐<br>Person 3 ☐ ☐<br>Person 4 ☐ ☐<br>Person 5 ☐ ☐
06b	If yes to Question 6: in the last 7 nights, how many nights did each person from Question 6 sleep under this net?	Person 1 ☐ night(s)<br>Person 2 ☐ night(s)<br>Person 3 ☐ night(s)<br>Person 4 ☐ night(s)<br>Person 5 ☐ night(s)	Person 1 ☐ night(s)<br>Person 2 ☐ night(s)<br>Person 3 ☐ night(s)<br>Person 4 ☐ night(s)<br>Person 5 ☐ night(s)	Person 1 ☐ night(s)<br>Person 2 ☐ night(s)<br>Person 3 ☐ night(s)<br>Person 4 ☐ night(s)<br>Person 5 ☐ night(s)

Interviewer Code: ________________

Endemicity-Village-Household ID: ☐☐☐☐☐☐

#	Bed Net Questions	Net #4	Net #5	Net #6
01	INTERVIEWER RESPONDS: Did you observe this net?	Observed 01<br>Not observed 00	Observed 01<br>Not observed 00	Observed 01<br>Not observed 00
02	INTERVIEWER RESPONDS: Are there holes in this net?	Yes 01<br>No 00<br>N/A 96	Yes 01<br>No 00<br>N/A 96	Yes 01<br>No 00<br>N/A 96
03	INTERVIEWER RESPONDS: Is this net correctly installed?	Yes 01<br>No 00<br>N/A 96	Yes 01<br>No 00<br>N/A 96	Yes 01<br>No 00<br>N/A 96
04	How many months have you had this net?<br>If <1 month, enter 00.<br>If >36 months, enter 95.<br>If don't know, enter 98.<br>If refused, enter 99.	☐☐ months	☐☐ months	☐☐ months
05	Where did you get this net?	Purchased at market 01<br>Mass distribution 02<br>Antenatal clinic 03<br>Hospital 04<br>N/A 96<br>Other 97<br>*Please list:* ________<br>Don't know 98<br>Refused 99	Purchased at market 01<br>Mass distribution 02<br>Antenatal clinic 03<br>Hospital 04<br>N/A 96<br>Other 97<br>*Please list:* ________<br>Don't know 98<br>Refused 99	Purchased at market 01<br>Mass distribution 02<br>Antenatal clinic 03<br>Hospital 04<br>N/A 96<br>Other 97<br>*Please list:* ________<br>Don't know 98<br>Refused 99
06	Did someone sleep under this net last night?<br>**IF NO: return to Question 1 for the next net or skip to page 7 if this is the last net.**	Yes 01<br>No 00<br>N/A 96<br>Don't know 98<br>Refused 99	Yes 01<br>No 00<br>N/A 96<br>Don't know 98<br>Refused 99	Yes 01<br>No 00<br>N/A 96<br>Don't know 98<br>Refused 99
06a	If yes to Question 6: who slept under this net?<br>Enter the name and age of each person.	Name / Age<br>Person 1 ☐ ☐<br>Person 2 ☐ ☐<br>Person 3 ☐ ☐<br>Person 4 ☐ ☐<br>Person 5 ☐ ☐	Name / Age<br>Person 1 ☐ ☐<br>Person 2 ☐ ☐<br>Person 3 ☐ ☐<br>Person 4 ☐ ☐<br>Person 5 ☐ ☐	Name / Age<br>Person 1 ☐ ☐<br>Person 2 ☐ ☐<br>Person 3 ☐ ☐<br>Person 4 ☐ ☐<br>Person 5 ☐ ☐
06b	If yes to Question 6: in the last 7 nights, how many nights did each person from Question 6 sleep under this net?	Person 1 ☐ night(s)<br>Person 2 ☐ night(s)<br>Person 3 ☐ night(s)<br>Person 4 ☐ night(s)<br>Person 5 ☐ night(s)	Person 1 ☐ night(s)<br>Person 2 ☐ night(s)<br>Person 3 ☐ night(s)<br>Person 4 ☐ night(s)<br>Person 5 ☐ night(s)	Person 1 ☐ night(s)<br>Person 2 ☐ night(s)<br>Person 3 ☐ night(s)<br>Person 4 ☐ night(s)<br>Person 5 ☐ night(s)

Interviewer Code: ________________

Baseline Malaria Diagnostic Results. *(To be completed for each subject.)*

Endemicity-Village-Household ID: | | | | | | |

Participant Name: ______ Participant Age: ______ Participant Number: | | |

BASELINE MALARIA QUESTIONS

1. In the last 6 months has the participant been diagnosed with malaria by the Health Center or Hospital?

No 00	Yes, once 01	Yes, more than once 02	Don't know 98	Refused 99

2. In the last 6 months, has the participant been treated with an antimalarial medicine?

No 00	Yes 01	Don't know 98	Refused 99

2a. If so, which one:

None 00	Artemisinin-based combination therapy (ACT): artesunate/amodiaquine, artémether/lumefantrine 05	Malaxin 07	N/A 96
SP/Fansidar 01		Malaritab 08	Other 97
Chloroquine 02		Arinate 09	Don't know 98
Amodiaquine 03	Other ACT: arsucam/ co-arinate, etc. 06	Artesunate 10	Refused 99
Quinine 04		Mefloquine 11	

If other, please list: ____________________

3. In the past week, has the participant had a fever?

No 00	Yes 01	Don't know 98	Refused 99

3a. If a thermometer was used, what was the temperature?

°C	N/A 96	Don't know 98	Refused 99

4. Is participant pregnant?

No 00	Yes 01	N/A 96	Don't know 98	Refused 99

4a. If yes to Q7, is an antimalarial being taken for malaria prevention?

No 00	Yes, SP (Fansidar) 01	Yes, other antimalarial 02	N/A 96	Don't know 98	Refused 99

If other, please list: ____________________

BLOOD TESTS

Collection date and time: |__|__| - |__|__| - |__|__|__|__| |__|__| : |__|__|
day month year hours minutes

5. Was a rapid diagnostic test performed?

No 00	Yes 01

If no, please state reason: ____________________

5a. If yes to Q5, rapid diagnostic test results:

Negative 00	Positive for *P. falciparum* 01	Positive for a species other than *P. falciparum* 02	Positive for mixed infection 03	Indeterminate 04	N/A 96

If indeterminate, please state reason: ____________________

5b. If positive, referred to Health Clinic?

No 00	Yes 01

If no, please state reason: ____________________

Interviewer Code: ____________

Baseline Malaria Diagnostic Results. *(Continued)*

Endemicity-Village-Household ID: | | | | | | |

Participant Name: ________ Participant Age: ____ Participant Number: | | |

6. Was a dried blood spot (DBS) collected?

No 00	Yes 01

If no, please state reason: ____________________________________

7. The filter paper must be labeled with the 8-digit code (household ID followed by subject number).

   I have checked to make sure the filter paper number is correct.

No 00	Yes 01

If no, please state reason: ____________________________________

SIGNATURES

Lab technician: ____

Interviewer: ____

Interviewer Code: _______________
