# Supplementary material for: Individual and household characteristics of persons with Plasmodium falciparum malaria in sites with varying endemicities in Kinshasa Province, Democratic Republic of the Congo
Source: Malar J. 2017 Nov 9;16:456. doi: 10.1186/s12936-017-2110-7 (PMC5680818; doi:10.1186/s12936-017-2110-7)
Supplement: Supplementary file 2 — Additional file 2: Table S1. PCR Primers. [file 12936_2017_2110_MOESM2_ESM.docx]

| **Target** | **Primer sequences (5’-> 3’)** | **Reaction conditions** | **Cycling parameters** | **Refs** |
| --- | --- | --- | --- | --- |
| *pfldh*  HumTuBB | For: ACGATTTGGCTGGAGCAGAT  Rev: TCTCTATTCCATTCTTTGTCACTCTTTC  Probe: FAM-AGTAATAGTAACAGCTGGATTTA  CCAAGGCCCCA-TAMRA    For: AAGGAGGTCGATGAGCAGAT  Rev: GCTGTCTTGACATTGTTGGG  Probe: VIC-TTAACGTGCAGAACAAGAACAGC  AGCT-TAMRA | 200nM each primer  100nM each probe  Probe Master qPCR Mix (Roche Diagnostics, Indianapolis, IN)  2μL template DNA  12 μL reaction vol | 50°C x 2min;  95°C x 10min;  40 cycles of 95°C x 15sec, 60°C x 1min | 1, 2 |

Abbreviations: MM, master mix. Vol, volume.

Table S1. Primers, probes, and reaction conditions for the duplex real-time PCR assay targeting the *P. falciparum*-specific *pfldh* gene and the human beta tubulin gene (HumTuBB).

1. Picard AL, *et al.* (2003) Resistance to Antimalarials in Southeast Asia and Genetic Polymorphisms in *pfmdr1. Antimicrobial Agents and Chemotherapy* 47(8):2418-2423.

2. Beshir KB*, et al.* (2010) Measuring the efficacy of anti-malarial drugs in vivo: quantitative PCR measurement of parasite clearance. *Malaria journal* 9(1):31
